# Diagnostic Performance of Point Shear Wave Elastography (pSWE) Using Acoustic Radiation Force Impulse (ARFI) Technology in Mesenteric Masses: A Feasibility Study

**DOI:** 10.3390/diagnostics12020523

**Published:** 2022-02-18

**Authors:** Amjad Alhyari, Christian Görg, Christoph Frank Dietrich, Svenja Kawohl, Ehsan Safai Zadeh

**Affiliations:** 1 Interdisciplinary Center of Ultrasound Diagnostics, University Hospital Giessen and Marburg, Philipps University Marburg, Baldingerstraße, 35033 Marburg, Germany; alhyari@med.uni-marburg.de (A.A.); svenja-kawohl@gmx.de (S.K.); ehsan_sz@yahoo.de (E.S.Z.); 2 Gastroenterology, Endocrinology, Metabolism and Clinical Infectiology, University Hospital Giessen and Marburg, Philipp University of Marburg, Baldingerstraße, 35033 Marburg, Germany; 3 Department Allgemeine Innere Medizin (DAIM), Kliniken Hirslanden Bern, Beau Site, Salem und Permanence, 3018 Bern, Switzerland; c.f.dietrich@googlemail.com

**Keywords:** ARFI elastography, ultrasound, mesentery, sclerosing mesenteritis, mesenteric mass

## Abstract

Purpose: To evaluate the diagnostic performance of ultrasound point shear wave elastography (pSWE) using acoustic radiation force impulse (ARFI) technology in different benign and malignant mesenteric masses (MMs). Methods: A total of 69 patients with MMs diagnosed from September 2018 to November 2021 were included retrospectively in the study. The inclusion criteria were (1) an MM over 1 cm; (2) valid ARFI measurements; and (3) confirmation of the diagnosis of an MM by histological examination and/or clinical and radiological follow-up. To examine the mean ARFI velocities (MAVs) for potential cut-off values between benign and malignant MMs, a receiver operating characteristics analysis was implemented. Results: In total, 37/69 of the MMs were benign (53.6%) and 32/69 malignant (46.4%). Benign MMs demonstrated significantly lower MAVs than mMMs (1.59 ± 0.93 vs. 2.76 ± 1.01 m/s; *p* < 0.001). Selecting 2.05 m/s as a cut-off value yielded a sensitivity and specificity of 75.0% and 70.3%, respectively, in diagnosing malignant MMs (area under the curve = 0.802, 95% confidence interval 0.699–0.904). Conclusion: ARFI elastography may represent an additional non-invasive tool for differentiating benign from malignant MMs. However, to validate the results of this study, further prospective randomized studies are required.

## 1. Introduction

The mesentery is a complex, contiguous, membranous peritoneal fold, which starts at the level of the aortic origin of the superior mesenteric artery (known as the root of the mesentery) and then extends in a fan-like fashion toward its insertion at the intestinal border, thus connecting the intestine (from the level of the duodenojejunal junction to the rectum) to the posterior abdominal wall [1]. Several pathologies can affect the mesentery, which could be benign or malignant, solid or cystic, and primary (originating within the mesentery) or secondary. In many cases, the definite diagnosis requires obtaining biopsies or even surgical exploration [1,2]. Many benign mesenteric pathologies (mesenteropathies) present as a mass on imaging, making it difficult to distinguish benign from malignant mesenteropathies [3,4,5,6,7,8].

Computed tomography (CT) is the most widely utilized imaging modality in the diagnosis of mesenteric masses (MMs). In addition, magnetic resonance imaging (MRI), positron emission tomography–computed tomography (PET-CT), and B-mode ultrasound (US) are used for evaluating MMs [2,3,9,10]. As a new trend in US imaging, contrast-enhanced ultrasound (CEUS) is used to assess perfusion patterns of various pathologies, including MMs [11,12,13,14,15,16,17,18,19,20]. Another new trend in US imaging is US elastography, which is a non-invasive tool for assessing tissue stiffness, with guidelines available for hepatic and non-hepatic applications [21,22,23,24,25,26]. Acoustic radiation force impulse (ARFI) is a type of point shear wave elastography (pSWE) that depends on the differences of acoustic wave propagation velocity in different tissues, determined by measuring the degree of displacement within a 0.5 to 1.0 cm box on the US screen, known as the “region of interest” (ROI) [27]. In 2019, the European Federation for Ultrasound in Medicine and Biology (EFSUMB) published guidelines on the use of elastography in non-hepatic organs [21]. However, to date, no guidance for the use of ARFI elastography in the mesentery is currently available. 

The aim of this study was to evaluate the feasibility and diagnostic performance of US ARFI elastography as a quantitative, non-invasive method for characterizing MMs of various etiologies.

## 2. Materials and Methods

This study included 80 consecutive patients with MMs detected on B-mode US between September 2018 and November 2021, who underwent an elastographic evaluation (ARFI) at our tertiary healthcare facility (university hospital). This study was approved by the local ethics committee (protocol code: EK_MR_09_04_20_görg_2) and conducted in accordance with the amended Declaration of Helsinki. Informed consent for the US examination was obtained from each patient.

The inclusion criteria were (1) an MM over 1 cm; (2) valid ARFI measurements; and (3) confirmation of the diagnosis of an MM by histological examination and/or clinical and radiological follow-up. In total, 11/80 patients (13.8%) with an MM were excluded due to the absence of diagnostic confirmation. Finally, data from 69 MMs were analyzed retrospectively. 

### 2.1. Ultrasound Examinations

All US and ARFI elastographic examinations were performed using a Siemens Acuson S2000, Acuson S3000, and Acuson Sequoia (Siemens Medical Solutions, Erlangen, Germany) by a German Society for Ultrasound in Medicine (DEGUM) Level III-qualified examiner (C.G., internal medicine) with more than 35 years of US experience [28]. With the patient lying supine, the curved linear-array transducer (6C1) was placed on the abdomen, and the whole mesentery was examined systematically for suspected areas. Focus and gain were adjusted as needed. The transducer was placed gently on the abdomen where the mesenteric thickening or mass could be easily visualized, and the depth was adjusted, bringing the mesentery to the center of the screen. Both the echogenicity (hypoechoic, isoechoic) and size of the MM (largest diameter in cm) were evaluated on B-mode US.

### 2.2. Acoustic Radiation Force Impulse Examinations 

The ROI (dimensions = 10 × 5 mm) was positioned on the US screen completely within the MM. For each measurement, the patient was asked to hold their breath in mid-expiration for at least 6 s. The measurement was displayed as velocity (m/s) on the upper corner of the screen. If the lesion moved while being measured, this single reading “shot” was considered invalid and was repeated. In the event of air superimposition, the patient was positioned carefully to enable an adequate measurement to be made. A total of 11 valid measurements were obtained for each ARFI study [29,30]. Both the depth of the measurement and the mean ARFI velocities (MAVs) were registered. In the case of measurements at different depths within the lesion, an average depth was calculated.

### 2.3. Statistical Analysis

Demographic and biometric data were expressed as mean values ± standard deviations (SDs). Statistical evaluation was performed on the categorical variable using Fisher’s exact test and on the continuous data using Mann–Whitney tests. The diagnostic performance was assessed using receiver operating characteristics (ROC) curves. Cut-offs between groups were examined for accuracy using the area under the ROC curve (AUROC) and the 95% confidence interval (CI). A *p*-value of <0.05 was defined as significant. The statistical analyses were performed using Excel (Microsoft 365 MSO; Microsoft Corporation, Redmond, WA, USA) and SPSS Version 26.0 statistics software (IBM, Armonk, NY, USA).

## 3. Results

### 3.1. Demographic and Clinical Data

Of the 69 study patients, 51 (73.9%) were male and 18 (26.1%) female. The mean age was 62 ± 14 years (range 21–91). The mean body mass index (BMI) was 27.4 ± 5.5 kg/m^2^ (range 13.9–45.1). The final diagnosis was malignant mesenteric mass (mMM) in 32/69 cases (46.4%) and benign mesenteric mass (bMM) in 37/69 cases (53.6%). An overview of all diagnostic entities is shown in Table 1. 

In 28/32 cases (87.5%), the diagnosis of an mMM was confirmed by histological examination of the MM. In the other 4/32 cases (12.5%), the diagnosis of an mMM was confirmed by radiological examinations of the mMM and a histological examination of a distant metastasis. 

Of the 37 bMMs, in a total of 17 cases (45.9%) the diagnosis of a bMM was confirmed by histological examination of the MM. In the remaining 20/37 cases (54.1%), the diagnosis of a benign lesion was confirmed based on clinical follow-up and/or a cross-sectional imaging (CT or MRI).

### 3.2. B-Mode Ultrasound Data 

Malignant mesenteric masses were hypoechoic in 29/32 cases (90.6%) and isoechoic in 3/32 cases (9.4%), whereas bMMs were hypoechoic in 11/37 cases (29.7%) and isoechoic in 26/37 cases (70.3%). Hypoechoic MMs were significantly more frequently associated with malignancy compared to isoechoic MMs (*p* < 0.001, Fisher’s exact test). The mean size of all 69 MMs was 6.34 ± 3.93 cm (range 1.5–22.0 cm); the size did not differ significantly between bMMs (5.35 ± 2.40 cm) and mMMs (7.48 ± 4.98 cm; *p* = 0.15, Mann–Whitney test). 

### 3.3. Acoustic Radiation Force Impulse Examinations

**Figure 1:**
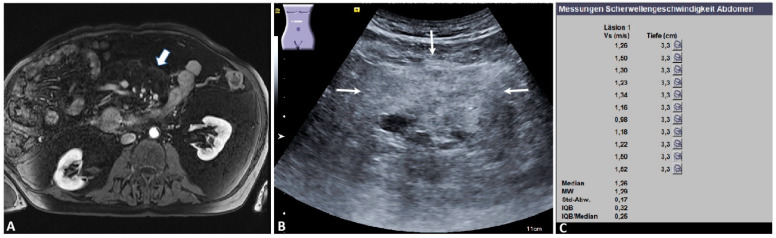
Benign mesenteric mass. An 80-year-old male patient with a known history of type 1 autoimmune pancreatitis under immunosuppressive treatment. (**A**) Magnetic resonance imaging appearance of a mass-like “misty mesentery” in the right upper portion of the umbilical region (arrow) (courtesy of Prof. Dr. Mahnken, Department of Radiology, University Hospital Marburg); (**B**) the ultrasound appearance of an ill-defined, slightly inhomogeneous, echogenic mesenteric mass (arrows); (**C**) the final acoustic radiation force impulse (ARFI) report of the same mass, showing a mean ARFI velocity (MW) of 1.29 m/s. The mesenteric histology showed an IgG−4-positive sclerosing mesenteritis. Läsion 1: lesion 1; Vs (m/s): velocity in meter per second; Tiefe (cm): depth in centimeter; MW = mean value (Mittelwert); Std-Abw.: standard deviation (Standard Abweichung); IQB: interquartile range (Interquartilbereich).

**Figure 2:**
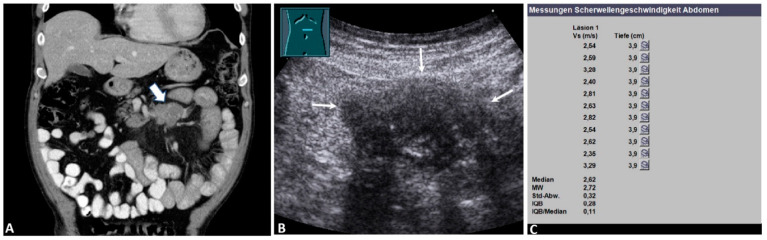
Malignant mesenteric mass. A 67-year-old male patient with a known history of malignant lymphoma and suspected recurrence on staging. (**A**) Computed tomography showing a hypointense round mass in the left upper quadrant (arrow) (courtesy of Prof. Dr. Mahnken, Department of Radiology, University Hospital Marburg); (**B**) B-mode ultrasound showing a hypoechoic mesenteric mass (arrows); (**C**) the final acoustic radiation force impulse (ARFI) report of the same mass, showing a mean ARFI velocity (MW) of 2.72 m/s. The mesenteric histology showed a high-grade malignant lymphoma. Läsion 1: lesion 1; Vs (m/s): velocity in meter per second; Tiefe (cm): depth in centimeter; MW = mean value (Mittelwert); Std-Abw.: standard deviation (Standard Abweichung); IQB = interquartile range (Interquartilbereich).

**Figure 3:**
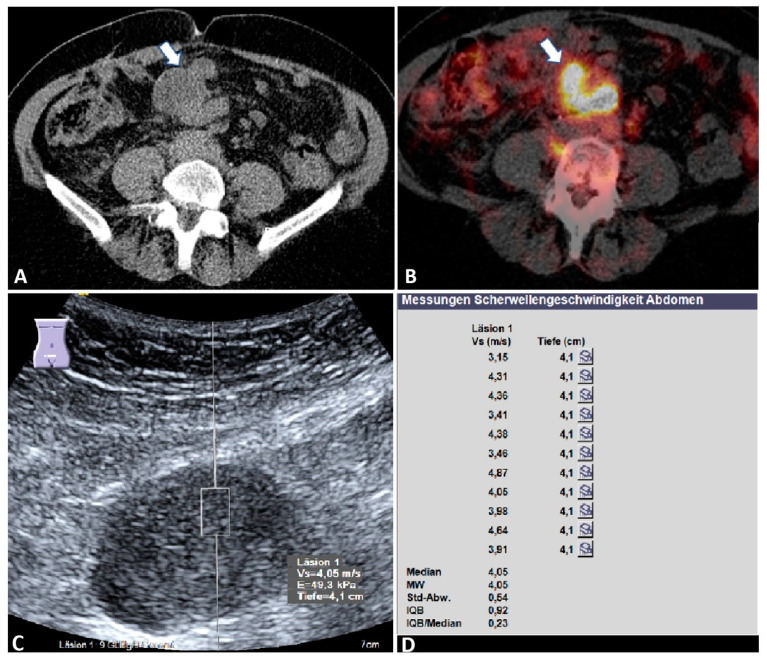
Malignant mesenteric mass. A 76-year-old female patient with a known history of ovarian cancer and suspected recurrence on staging. (**A**) Computed tomography image (left) shows a hypointense mesenteric mass (arrow) (courtesy of Prof. Dr. Mahnken, Department of Radiology, University Hospital Marburg); (**B**) positron emission tomography–computed tomography reveals a high intensity of fluorodeoxyglucose uptake within the mass (arrow)), indicating mesenteric metastasis (courtesy of Prof. Dr. Luster, Department of Nuclear Medicine, University Hospital Marburg); (**C**) B-mode ultrasound showing a hypoechoic mesenteric mass; (**D**) the final acoustic radiation force impulse (ARFI) report of the same mass, showing a mean ARFI velocity (MW) of 4.05 m/s. Läsion 1: lesion 1; Vs (m/s): velocity in meter per second; Tiefe (cm): depth in centimeter; MW = mean value (Mittelwert); Std-Abw.: standard deviation (Standard Abweichung); IQB = interquartile range (Interquartilbereich); E: elasticity in Kilopaskal (kPa).

**Figure 4:**
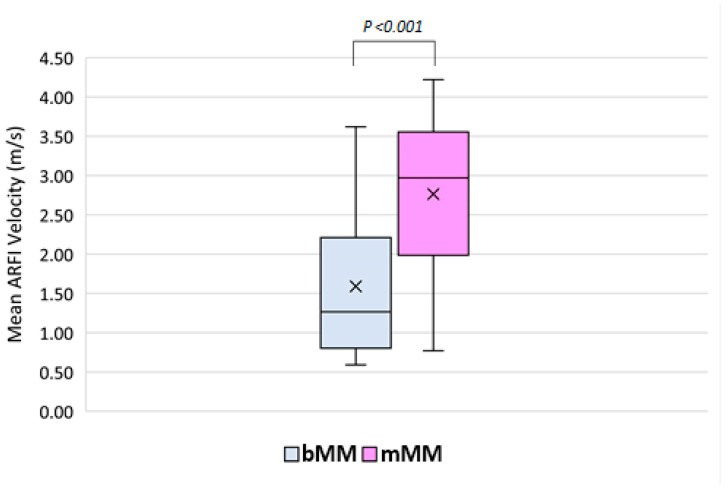
Differences in mean acoustic radiation force impulse (ARFI) velocities between benign and malignant mesenteric masses in the study. The mean ARFI velocity (MAV) in m/s is represented with an “X” in each box, and the median ARFI velocity in subgroups is shown as a horizontal line within each box. bMM: benign mesenteric mass (MAV = 1.59 ± 0.90 m/s), mMM: malignant mesenteric mass (MAV = 2.76 ± 1.01 m/s) (*p* < 0.001).

**Figure 5:**
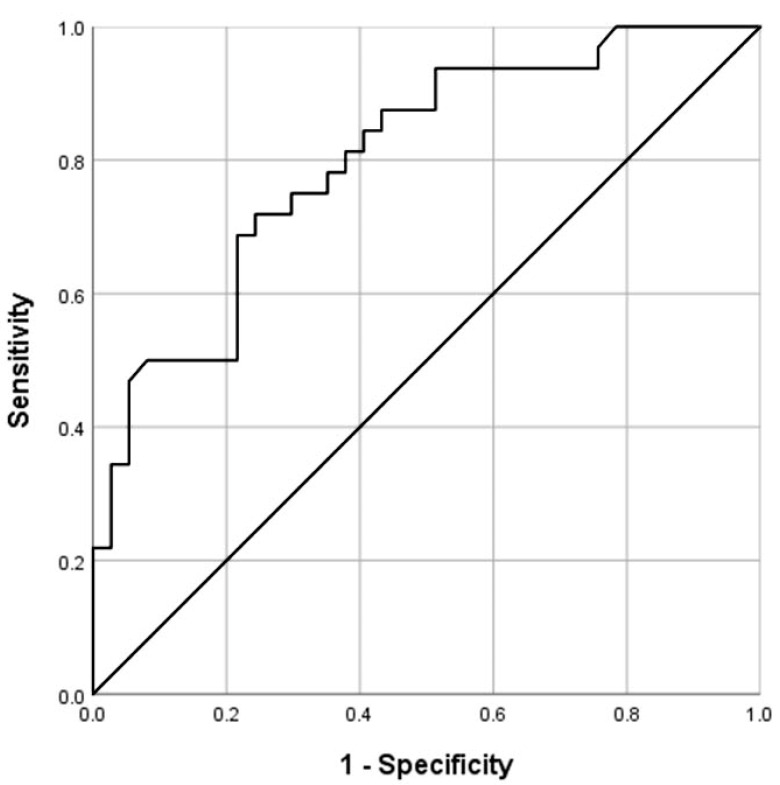
Receiver operator characteristic curve for the differences in mean acoustic radiation force impulse velocities between benign and malignant mesenteric masses.

Benign MMs demonstrated significantly lower mean MAVs than mMMs (1.59 ± 0.93 vs. 2.76 ± 1.01 m/s; *p* < 0.001, Mann–Whitney U-test; Figure 1, Figure 2, Figure 3 and Figure 4). The selection of 2.05 m/s as a cut-off value yielded a sensitivity, specificity, positive predictive value, and negative predictive value of 75.0%, 70.3%, 68.6%, and 76.5%, respectively, in diagnosing mMMs (area under the curve (AUC) = 0.802, 95% CI = 0.699–0.904, Figure 5) (Table 2). In subgroup analysis, hematological mMMs (15/32; 46.9%) showed high MAVs (2.73 ± 1.03 m/s), similar to those of non-hematological mMMs (17/32; 53.1%) (2.79 ± 1.03 m/s). Furthermore, the MAVs of sclerosing mesenteritis cases (15/69; 21.7%) (1.53 ± 0.96 m/s) were significantly lower than those of mMMs (32/69; 46.4%) (2.76 ± 1.01 m/s; *p* = 0.001). The MAVs among benign and malignant subgroups of MMs are shown in Table 2.

## 4. Discussion

The mesentery is one of the forgotten abdominal structures. The clinical, pathophysiological, and immunological functions of the mesentery as a distinct organ have been discussed increasingly in many recent reviews [1,31]. The clinical manifestation of mesenteropathies is variable [1,3]. Furthermore, some benign entities, such as mesenteritis, show similar radiological characteristics to those of malignant neoplasms, and distinction between these lesions and neoplastic lesions of the mesentery can present a radiological challenge [3]. Therefore, the need remains to develop new non-invasive methods to evaluate MMs. 

Shear wave elastography (SWE) uses the acoustic stimulation of tissue, which induces shear waves that propagate perpendicularly to the compressive (stimulating) signal; these waves are registered at different locations, allowing the estimation of their velocity, which in turn represents tissue elasticity [32]. There are two main types of SWE. In two-dimensional (2D)-SWE, body tissues are stimulated at different points, generating propagating shear waves, which are monitored in real time at different locations within the image, thus generating a quantitative elastogram in the form of a “topographic” colored map correlating to a scale of different elastic measurements in m/s or kilopascals [32]. In point shear wave elastography (pSWE), such as acoustic radiation force impulse (ARFI) pSWE, the resulting shear waves propagate in a direction perpendicular to the axial stimulating acoustic beam, and the speed of propagation of these shear waves is measured within a region of interest (ROI) by estimating the time needed to travel from the border near to the stimulus to the border away from the stimulus [33]. Both pSWE and 2D-SWE have excellent performance in assessing liver fibrosis and cirrhosis [34,35,36], with good reproducibility [34,37], and both modalities perform similarly well in differentiating malignant and benign lesions [38,39,40]. The feasibility of performing examinations in ascites and obesity is considered among the advantages of pSWE. Due to these features, ARFI elastography may be a suitable method for the evaluation of intra-abdominal pathologies. In a standardized study, we examined the performance of pSWE using ARFI as a new US-based sonographic modality in different MMs and as a potential tool in the diagnosis of different MMs. In this study, the mean ARFI values in the mMM group were 2.76 m/s with an SD of ±1.01 m/s (Figure 2 and Figure 3), and in the bMM group, they were 1.59 m/s with an SD of ±0.93 m/s (Figure 1). Malignant MMs demonstrated significantly higher MAVs compared with bMMs (*p* < 0.001, Mann–Whitney U-test). Using the AUROC and in order to maximize sensitivity and specificity, we suggest a value of 2.05 m/s as a cut-off for mMMs (AUC = 0.803, 95% CI 0.702–0.904). The calculated sensitivity, specificity, positive predictive value, and negative predictive value were 75.0%, 70.3%, 68.6%, and 76.5%, respectively. 

These findings reveal that pSWE using ARFI may be an additional useful non-invasive tool for the evaluation of malignancy of MMs in addition to B-mode US and CEUS (Table 3). Particularly in incidentally detected mesenteric pathologies, ARFI combined with B-mode US features and perfusion patterns on CEUS may help in developing diagnostic algorithms. However, in the presence of malignant disease or clinical suspicion of malignant disease, PET-CT remains the imaging method with the highest diagnostic performance (Table 3).

A further notable finding of this study was the difference between the MAVs of sclerosing mesenteritis and those of mMMs. The MAVs of sclerosing mesenteritis (15/69; 21.7%) (1.53 ± 0.96 m/s) were significantly lower than those of mMMs (32/69; 46.4%) (2.76 ± 1.01 m/s; *p* = 0.001). These results are important because sclerosing mesenteritis and mMMs may have similar features in different imaging modalities, such as CT or CEUS, and may not be differentiated from each other [11,41]. However, it should be noted that sclerosing mesenteritis may also be caused secondarily due to an underlying malignant disease [42]. Therefore, histological confirmation should always be strongly considered when sclerosing mesenteritis is suspected. The MAVs of hematologic malignancies (15/69; 21.7%) (2.79 ± 1.03 m/s) were not significantly different compared with those of non-hematologic malignancies (17/69; 24.6%) (2.73 ± 1.03 m/s; *p* > 0.05), and a differentiation between hematologic and non-hematologic malignancies by ARFI elastography was not possible. 

There were some limitations to this study. (1) Technical difficulty and failure of measurement: due to the anatomical location of the mesentery and the superimposition of air-filled intestinal loops, ARFI examination of the mesentery may be difficult. In the event of air superimposition, the patient was positioned carefully to enable an adequate measurement to be made. (2) Reproducibility: due to the retrospective nature of the study, the evaluation of inter-rater reliability in the performance of the ARFI elastography investigation in the mesentery was not possible; however, the reproducibility of ARFI has been described in various studies in other organs [30,37,43]. (3) Small sample size: due to the relatively small number of subjects (*n* = 69), further large prospective studies are needed to validate these results. (4) Validation of diagnosis: a histological examination was not performed in all the MMs. However, all the diagnoses in these patients were confirmed by a future cross-sectional imaging modality (CT or MRI) and clinical follow-up.

## 5. Conclusions

Our understanding of the mesenterial organ in terms of structure, composition, and role in health and disease is still evolving. In this study, we confirmed the feasibility of pSWE using ARFI in quantifying the degree of stiffness of different benign and malignant mesenteric pathologies. We also found significant differences in stiffness between bMMs and mMMs. Elastography, in particular pSWE, may represent a helpful diagnostic modality in the evaluation of mesenteric pathologies. However, to validate the results of this study, further prospective randomized studies are required.

## Figures and Tables

**Table 1:** Overview of all diagnostic entities in the study sample (*n* = 69).

MM Subgroup	Malignant Hematological MM	Malignant Non-Hematological MM	Benign Inflammatory MM (Mesenteritis)	Mesenteric Lipomatosis	Other Benign Masses
**No. of lesions**	(*n* = 15)	(*n* = 17)	(*n* = 26)	(*n* = 8)	(*n* = 3)
**Etiology**	- Lymphoma (14) - Chloroma (1)	- Desmoid fibromatosis (2) - GIST (3) - CRC (2) - NET (3) - PDAC (1) - Liposarcoma (2) - Merkel cell carcinoma (1) - Gastric carcinoma (1) - Follicular thyroid carcinoma (1) - Ovarian carcinoma (1)	- Sclerosing mesenteritis (15) - Mesenteric involvement in Crohn’s disease (4) - Fat necrosis in necrotizing pancreatitis (2) - Mesenteritis in acute edematous pancreatitis (1) - Mesenteritis due to perforated bowel (2) - Non-specific (2)	- Mesenteric fat bulk (8)	- Heterotopic pancreas (2) - Lipoma (1)

CRC: colorectal carcinoma, GIST: gastrointestinal stromal tumor, MM: mesenteric mass, NET: neuroendocrine tumors, PDAC: pancreatic ductal adenocarcinoma. The number between brackets () is the total number of patients in each category.

**Table 2:** Comparison of acoustic radiation force impulse (ARFI) data in different benign and malignant mesenteric masses in *n* = 69 study patients.

Subgroup	Number of Lesions (n)	ARFI Velocity (m/s)			Average Depth of Measurement (Mean ± SD in cm)
		Mean ± SD	Minimum	Maximum	
**bMMs**	37	1.59 ± 0.93	0.59	3.62	4.75 ± 1.20
Mesenteritis	26	1.75 ± 1.02	0.59	3.62	4.53 ± 0.99
Mesenteric fat bulk	8	0.90 ± 0.24	0.67	1.35	5.43 ± 1.65
Other benign masses	3	1.99 ± 0.13	1.86	2.12	4.83 ± 1.26
**mMMs**	32	2.76 ± 1.01	0.77	4.22	4.25 ± 0.80
Non-hematological	17	2.79 ± 1.03	1.18	4.22	4.07 ± 0.80
Hematological	15	2.73 ± 1.03	0.77	4.18	4.47 ± 0.78

ARFI: acoustic radiation force impulse, bMM: benign mesenteric mass, mMM: malignant mesenteric mass, SD: standard deviation.

**Table 3:** Comparison of various imaging modalities regarding their diagnostic performance in differentiating benign and malignant mesenteric masses.

Author	Number of Patients	Imaging Modality	Parameter Predictive of Malignancy	Sensitivity (%)	Specificity (%)	PPV (%)	NPV (%)	*p*-Value
Trenker et al., (2017) [11] *	69	B-mode US	Hypoechoic or complex echogenicity	94	50	80	79	<0.001
			Regular borders	81	68	84	63	<0.05
Trenker et al., (2017) [11] *	69	CEUS	Parenchymal phase washout	75	59	80	52	<0.05
Nakatani et al., (2013) [12] **	71	PET-CT	Diam_max_ > 10 mm	69	98	90	93	<0.001
			SUV_max_ ≥ 3.0	85	98	92	96	<0.001
**Present study**	69	ARFI elastography	Mean velocity > 2.05 m/s	75	70	69	77	<0.001

CEUS: contrast-enhanced ultrasound, Diammax: maximum short-axis diameter of mesenteric masses, NPV: negative predictive value, PET-CT: positron emission tomography–computed tomography, PPV: positive predictive value, SUVmax: maximum standardized uptake value, US: ultrasound. * According to the results of this study, we calculated the sensitivity, specificity, PPV, and NPP for B-mode US and CEUS. ** This study examined the diagnostic performance of Fludeoxyglucose-PET-CT in distinguishing viable malignant lesions from benign conditions in patients with misty mesentery (patients with bulky mesenteric masses were excluded).

## Data Availability

The data presented in this study are available on request from the corresponding author.
